# Effect of classroom physical activity breaks on learning behaviors and academic achievements in university students: a systematic review

**DOI:** 10.1186/s12966-026-01936-7

**Published:** 2026-06-20

**Authors:** Qianting Deng, Chongyun Wu, Luodan Yang

**Affiliations:** Laboratory of Exercise and Neurobiology, School of Physical Education and Sports Science, South China Normal University, Guangzhou, 510006 China

**Keywords:** Classroom physical activity breaks, Student, Attention, Engagement, Emotion, Academic performance

## Abstract

**Background:**

College students’ prolonged sedentary behavior due to heavy academic burdens impairs cognitive functions and academic performance. Classroom physical activity breaks have been studied in younger students, but evidence among college students remains limited. This study aims to systematically evaluate the effects of classroom physical activity breaks on academic-related outcomes among college students, including classroom engagement, academic emotions, attention, and academic performance.

**Methods:**

A comprehensive literature search was conducted across databases, including PubMed, Web of Science, and ERIC (EBSCOhost), covering all publications up to May 2025, supplemented by manual search and citation tracking.

**Results:**

A total of 5,813 records were initially identified, and 20 studies met the inclusion criteria. Overall, classroom physical activity breaks were positively associated with improvements in sustained attention, emotional engagement, and positive academic emotions. However, findings for selective attention, behavioral engagement, negative emotions, and academic performance were mixed or inconclusive, with several studies reporting non-significant effects and one study reporting a negative effect on selective attention.

**Conclusions:**

Physical activity breaks in the classroom improved students’ learning behaviors and may also contribute to enhanced academic outcomes. This study highlights that, provided teaching efficiency is not compromised, the integration of classroom physical activity breaks represents a viable and impactful educational intervention.

**Supplementary Information:**

The online version contains supplementary material available at https://doi.org/10.1186/s12966-026-01936-7.

## Background

Sedentary behavior, characterized by wakefulness with low energy expenditure (≤ 1.5 METs), is closely associated with various adverse health outcomes [1]. It increases the risk of chronic non-communicable diseases and all-cause mortality, diminishes positive affect, and increases susceptibility to depression and anxiety [2, 3]. In addition, prolonged sitting detrimentally affects metabolic health and may impair cognitive performance and overall brain health. Numerous studies have demonstrated a significant decline in physical activity levels from adolescence to early adulthood, leading to a predominantly sedentary lifestyle in later years [4, 5]. In particular, academic activities in higher education predominantly occur in sedentary environments, often characterized by prolonged sitting with minimal interruptions [6, 7]. Studies have indicated that university students spend substantial amounts of time engaged in sedentary behavior, such as attending lectures, reading, or performing computer-based tasks, with average daily sedentary time often approaching or exceeding 10 h per day [8]. Despite the prevalence of sedentary behavior, prolonged sitting in university environments has garnered relatively limited attention, likely attributed to entrenched cultural perceptions that regard sitting as an appropriate and even essential posture for knowledge acquisition and contemplation [9, 10]. Notably, sedentary behavior affects the students’ current health status, their academic performance, and their overall learning outcomes [11]. Consequently, to mitigate the health risks associated with a sedentary lifestyle, public health guidelines in several countries emphasize the importance of reducing sedentary time through increased physical activity and regular interruptions of prolonged sitting [12].

In the context of education, multiple studies have explored the incorporation of classroom-based physical activity breaks as a strategy to enhance student physical activity without compromising instructional time [13, 14]. These interventions have been reported to improve perceived readiness to learn, emotional regulation, and several classroom-related outcomes [14, 15]. Additionally, research indicates that up to 95% of university students prefer opportunities to stand during class, and over half believe that active breaks contribute to improving their health, enhancing concentration, and alleviating discomfort [16]. Compared with uninterrupted sitting, regular physical activity breaks may be associated with improved affective states, reduced fatigue, and greater student receptivity [13, 17]. Interventions focusing on classroom physical activity breaks refer to structured physical activities implemented during standard academic lessons (excluding physical education), through brief movements or by integrating physical tasks into instructional content [18, 19]. Classroom physical activity breaks strategies may take several forms [19], including active breaks, curriculum-aligned activities, and movement-based learning. In addition to short active breaks, the implementation of sit-stand desks has emerged as a complementary strategy, enabling students to alternate between sitting and standing without disrupting classroom instruction [15]. Although concerns have been raised regarding potential disruption to instructional flow, the available observational and self-reported evidence suggests that such interventions are generally feasible and acceptable in classroom settings [20]. However, whether they consistently translate into measurable academic benefits remains uncertain.

Given the well-documented health risks associated with sedentary behavior among university students, targeting interventions within academic settings is of critical importance. Accordingly, the present systematic review aims to examine the impact of classroom-based physical activity breaks interventions on academic-related outcomes in university students, focusing specifically on classroom attention, engagement, academic emotions, and academic performance.

## Methods

### Search strategy

This systematic review was conducted across Web of Science, ERIC (EBSCOhost), and PubMed from inception to May 2025 to identify studies on the effects of classroom physical activity breaks on college students’ learning behaviors and academic performance. Details of the search strategy are provided in the supplementary Table 1. Search terms included intervention (e.g., classroom physical activity), population descriptors (e.g., college), and outcome-related keywords (e.g., attention, engagement, emotion, and academic performance). Related definition, see Table 1.

**Table 1:** Definitions of research term

Term	Definition	Reference
Classroom physical activity breaks	Structured physical activities implemented during standard academic lessons, including active breaks, curriculum-aligned activities, and movement-based learning	[18]
Active breaks	Brief interruptions of academic content to engage in physical movements unrelated to the instructional material	[19]
Curriculum-aligned activities	Embedding subject-specific content into short exercise tasks, allowing students to learn while being physically active	[19]
Movement-based learning	Deeply integrating physical activities into the teaching process, becoming an integral component of academic instruction.	[19]
Classroom attention	The ability to focus on relevant stimuli while suppressing distractions, including selective attention and sustained attention	[62]
Selective attention	The capacity to actively select and process specific information from multiple inputs while suppressing irrelevant stimuli	[63]
Sustained attention	The ability to maintain focus over extended periods	[64]
Classroom engagement	Students’ active involvement in learning tasks, including behavioral, emotional, cognitive, and agentic engagement	[65]
Behavioral engagement	Students’ observable involvement in academic tasks, including proactive retrieval of learning materials, and active communication and interaction	[66].
Emotional engagement	Students’ affective involvement and attitudinal orientation toward classroom learning, encompassing motivation and a sense of classroom belonging	[67].
Cognitive engagement	The use of higher-order learning strategies to enhance academic understanding and retention	[68]
Agentic engagement	Students proactively enrich the learning environment by constructively influencing instructional processes.	[69]
Academic emotions	Affective states (positive or negative) experienced in academic settings	[70]
Academic performance	Denotes measurable learning outcomes, typically assessed through examinations or academic tasks.	[71].

### Eligibility criteria

Two independent reviewers (QD and CW) screened the titles and abstracts to identify studies that met the following inclusion criteria: [1] targeted healthy college students; [2] implementation of classroom-based physical activity interventions; [3] assessed at least one academic-related outcome; [4] adopted an intervention study design, including randomized or non-randomized controlled trials, quasi-experimental studies, crossover studies, or single-arm intervention studies; and [5] were peer-reviewed full-text articles published in English. Exclusion criteria included reviews, editorials, technical reports, dissertations, and unpublished works. Any disagreements between reviewers were resolved through discussion or adjudication by a third reviewer (LY) when necessary.

### Study selection and data extraction

**Fig. 1:**
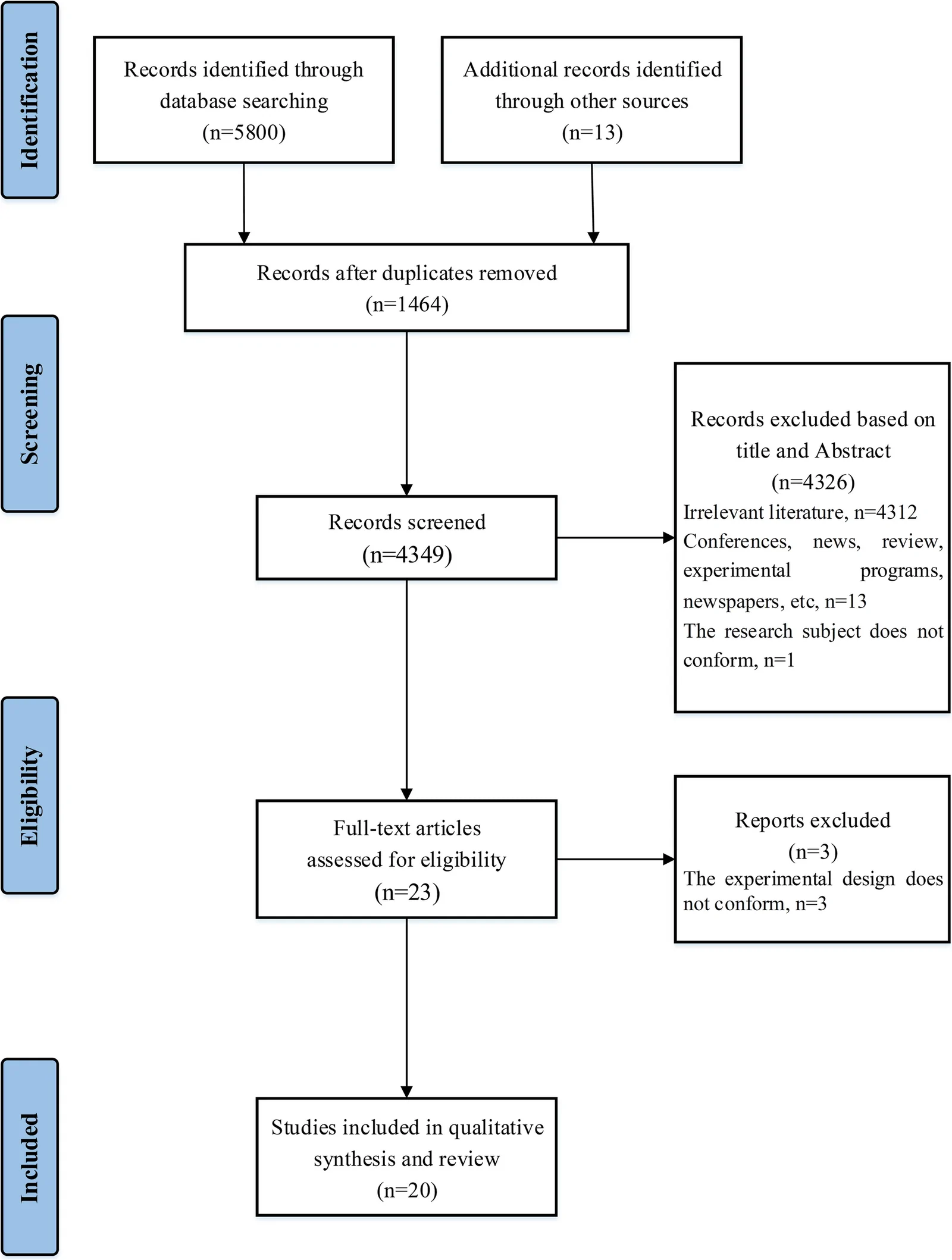
PRISMA flow diagram indicating the selection process for studies through the stages of the systematic review

This review followed PRISMA guidelines and was registered with PROSPERO (CRD420251047553). The completed PRISMA 2020 checklist is provided in Supplementary Table 2. Of 5,813 articles retrieved, 1,464 duplicates were removed. Titles and abstracts of 4,349 articles were screened, and 23 articles underwent full-text review. Finally, 20 articles met the inclusion criteria (Fig. 1). Extracted data included author, year, sample characteristics, design, comparison groups, intervention features, and outcome. Screening and selection were conducted independently by two reviewers (QD and CW), with disagreements resolved by discussion or third-party adjudication (LY).

### Quality assessment and certainty of evidence

Two reviewers (QD and CW) independently assessed methodological quality using the Downs and Black checklists, which evaluate reporting (items 1–10), external validity (items 11–13), internal bias (items 14–20), confounding (items 21–26), and power (item 27). However, item 27 (statistical power) was scored dichotomously: 1 if present, 0 if absent. Studies were classified as excellent (26–28], good (20–25], moderate (15–19], or poor (≤ 14). Furthermore, the GRADE approach was utilized to evaluate the strength of the evidence. Certainty ratings were based on considerations of risk of bias, inconsistency, indirectness, imprecision, and publication bias. Discrepancies were resolved through discussion, with a third reviewer (LY) consulted if consensus could not be reached.

### Coding and classification of outcomes

Considering the heterogeneity of the included studies, outcomes were coded as “**+**” (significant positive), “**-**” (significant negative), or “n.s.” (non-significant). For outcomes reported in three or more studies, evidence strength was rated as: “‡” indicates strong evidence (66.8%-100%), “?” indicates inconclusive evidence (33.4%-66.7%), “†” indicates weak evidence (0-33.3%). This coding framework was informed by prior work and operationalized for the present review [21].

## Results

### Study characteristics

This review synthesizes empirical evidence on whether classroom physical activity breaks have a positive effect on college students’ learning behaviors (classroom attention, engagement, and emotional states) and academic performance. A total of 20 studies were included, comprising 8,174 college students aged 18–25 years. The studies were conducted across nine countries, with the largest proportion originating from the United States (35.0%), followed by Canada (15.0%). Australia, Germany, and Austria each contributed two studies (10.0%), whereas France, Ireland, China, and Spain each contributed one study (5.0%). In terms of study design, the included studies comprised two randomized controlled trials, five controlled experimental studies, six quasi-experimental studies, six feasibility/pilot studies, and one single-arm study. Among the included studies, ten studies employed short bouts (inserting or integrating) of exercise in the classroom, typically two to three sessions of 5 to 10 min per class [13, 14, 17, 22–28]. Additionally, ten studies introduced dynamic learning equipment, such as standing desks, cycling desks, and balance balls, to replace traditional static seating (29–38]. However, notable variation existed in the frequency and duration of the interventions, ranging from single-session applications to repeated interventions lasting up to 10 to 15 weeks. In terms of measurement indicators, attention was the most frequently assessed outcome, followed by classroom engagement, emotional state, and academic performance. Attention was mainly evaluated using standardized tools, while engagement and emotions were assessed using self-reported questionnaires and affective scales. Academic performance was measured using test scores, self-reported grades, and the quality of task completion. A summary of the basic characteristics of all included studies is presented in Table 2.

**Table 2:** Characteristics of the selected studies

Study	Countries	Sample (size and age)	Study design	Intervention type	Outcome measures	Main result
[29]	Australia	*N* = 45 ♂ = 22 ♀ = 13 Age: 22.67 ± 6.27	RCT	Intervention: Sit-stand workstation or Walking workstation (1–3 km/hr) Control: Sitting condition	Attention assessment: Stroop Color Word Test and Paced Auditory Serial Addition Task	No significant change in cognitive performance and attention
[35]	United States	*N* = 21 ♂ = 6 ♀ = 15 Age: 19–24	RCT	Intervention: Stationary cycle desks (*N* = 9): participants were instructed to pedal at a low RPE of ≤ 2 during 50-minute lectures 3 times per week for 12 weeks. Control (*N* = 12): Sat stationary at traditional desks	Academic performance assessment: examination	No significant differences in test scores
[22]	Germany	*N* = 528 ♂ = NA ♀ = NA Age: NA	QES	Intervention: Standing Break Group (*N* = 304): stand up for a 5-minute break during 90-minute lectures Active Break Group (*N* = 102): 5-minute structured exercises during lectures. Control: Open Break Group (*N* = 122): stay seated or move around	Attention engagement and emotion assessment: questionnaires (four-point Likert scale)	Exercise breaks enhanced muscle relaxation (> 75%), concentration (> 75%), motivation (> 80%), and well-being (> 90%) in students Compared with open break, the standing and active break significantly improved well-being An active break specifically elevated learning motivation
[14]	Australia	*N* = 85 ♂ = 26 ♀ = 58 Age: 23 ± 2	F/P	Intervention: 5 to 10 min of whole-body movement breaks were scheduled after every 20 min of sedentary time in a 2-hour class Control: Seated throughout the 2-hour class	Attention and emotion assessment: visual analogue scale	Higher levels of concentration and enjoyment after exercise breaks (*p* < 0.001)
[38]	United States	*N* = 117 ♂ = 26 Age: 18.33 ± 0.72 ♀ = 58 Age: 18.39 ± 0.94	QES	Intervention (*N* = 59): Stationary bike workstation with a desktop for a minimum of 2 h per week over 10 weeks. Control (*N* = 58): Traditional desk	Emotion assessment: five-item Morale Scale Engagement assessment: six-question scale Academic performance assessment: 5-point scale	No significant differences in academic performance, morale score and engagement The student reported that studying at a traditional desk was easier.
[25]	United States	*N* = 223 ♂ = NA ♀ = NA Age: 18–23	F/P	Intervention: Student-led two 5-minute exercise breaks during an 80-minute lecture (twice per week for 15 weeks). Control: Traditional classroom	Attention, engagement, and emotion assessment: open-ended comments	Exercise breaks enhanced attention, increased course enjoyment, promoted interaction and engagement (*P* < 0.001)
[34]	United States	*N* = 304 ♂ = NA ♀ = NA Age: 20.1 ± 1.3	QES	Intervention: Sit-stand desk Control: Traditional fixed-seated desk	Attention, engagement, and emotion assessment: questionnaires	Students reported sit-stand desk enhanced classroom engagement (34.7%), concentration (50.5%), interaction (36.6%), and reduced negative emotions (42.6%-53%)
[30]	Canada	*N* = 99 ♂ = 43 ♀ = 51 ⚦ = 5 Age: 28.3 ± 9.8	QES	Intervention: Portable pedal exercise machine (DeskCycle™) or Adjustable-height standing desk (ProPlus 36™) Control: Conventional sitting desk	Academic performance assessment: questionnaire	Computer tasks were preferred on the standing desk (*P* = 0.001) Paperwork tasks preferred on the portable pedal exercise machine (*P* = 0.037)
[72]	France	*N* = 663 ♂ = 62.9% ♀ = 37.1% Age: 18.7 ± 1.6	F/P	Intervention: Standing Desks, Swiss Balls, Cycling Desks, and Pedal- or Stepper-Boards Control: Traditional Workstations	Attention, engagement, and emotion assessment: questionnaires	Student reported that reduced fatigue (34.4%), relieved stress (40.2%), decreased boredom (57.4%), and reduced off-task (35.2%) after using active workstations No significant differences in attention and engagement
[17]	Austria	*N* = 66 ♂ = 13 ♀ = 53 Age: 22.5 ± 5.5	CES	Intervention: Unstructured Break: participants remained seated at their desks for 6 min Exercise Breaks: 3 min of aerobic exercise and 3 min of stretching exercises Relaxation Break: 6 min of whole body relaxation exercise Control: continued the 4-hour lecture without any rest break	Emotion assessment: questionnaires (Eigenzustandsskala)	Structured exercise breaks enhance classroom vitality and reduce fatigue
[31]	United States	*N* = 79 ♂ = NA ♀ = NA Age: NA	QES	Intervention: Standing Desks, Treadmill Desks, and Balance-Ball Chairs Control: Seated Desks	Engagement assessment: questionnaires	Students (45.6%) believe that the active workstation improves learning ability.
[36]	United States	*N* = 79 ♂ = NA ♀ = N Age: 26.2 ± 10.2	F/P	Intervention: Portable pedal exercise machine (MagneTrainer) Control: Traditional seat	Engagement assessment: questionnaires	Students (87.5%) reported acceptability and usability of pedal machines
[37]	Germany	*N* = 2809 ♂ = 67% ♀ = 33% Age: NA	QES	Intervention: using sit-stand desks for 2 weeks Control: Traditional seat	Performance assessment: SOPLAY	Sit-stand desks reduce sedentary behavior and increase the standing rate
[13]	Ireland	*N* = 106 ♂ = 19 ♀ = 87 Age: NA	F/P	Intervention: followed a 4-minute active break video Control: continued sitting	Engagement assessment: questionnaires	Students (93.6–96.2%) recognize the value of exercise breaks
[26]	Austria	*N* = 51 ♂ = 34 ♀ = 17 Age: 22.3 ± 2.0	CES	Intervention (*N* = 26): Physical activity group: 10-minute outdoor running during the break of a 2-hour lecture Control (*N* = 25): remained sedentary	Attention and emotion assessment: questionnaire	Intervention in study breaks enhanced visual attention and affective states (pleasure)
[28]	China	*N* = 71 ♂ = 35 ♀ = 36 Age: 20.94 ± 1.93	CES	Intervention: Moderate-intensity cycling: Insert 15 min of cycling (60%VO_2_peak) in 100 min class High-intensity cycling: Insert 15 min cycling (80%VO_2_peak) in 100 min class Control: Uninterrupted sitting in 115 min class	Engagement and academic performance assessment: The Modified Dual-Task Stroop Task	Exercise breaks shorten reaction time and reduce the error rate (*P* < 0.05)
[32]	Canada	*N* = 24 ♂ = NA ♀ = NA Age: 23 ± 3.6	CES	Intervention: light-intensity cycling (RPE 11–13) or moderate-intensity cycling (70% heart rate reserve) while watching the documentary Control: remained seated for 30 min	Attention assessment: the eye tracker Emotion assessment: POMS-SF scale Academic performance assessment: examination	The visual attention of the moderate-intensity cycling group was lower than that of the other group (*P* < 0.001) The perceived workload of the moderate-intensity cycling group was higher than that of the other group(*P* < 0.001) The examination score of the moderate-intensity cycling group was lower than that of the low-intensity group (*p* = 0.009)
[24]	United States	*N* = 53 ♂ = NA ♀ = NA Age: NA	SA	Intervention: activity break every 15–20 min in class (randomly selected exercise items)	Emotion assessment: questionnaires	Students (66%) reported that physical activities break increases learning pleasure and concentration.
[27]	Spain	*N* = 26 ♂ = 12 ♀ = 14 Age: 23.36 ± 1.98	F/P	Intervention: After a 25-minute class, participants performed 10 min of physical activity (60–80%VO_2max_) Control: After the 25-minute class, participants engaged in 10 min of passive rest (reading a scientific paper)	Attention assessment: Psychomotor vigilance task	The psychomotor vigilance task response of the experimental group was significantly faster than that of the control group (*P* < 0.01)
[23]	Canada	*N* = 77 ♂ = 12% ♀ = 78% Age: 18.7 ± 1.4	CES	Intervention: three 5-minute exercise breaks during the 50-minute lecture Control: Play computer games for 5 min during each break continued sitting	Attention assessment: questionnaires, Academic performance assessment: examination (Immediate and after 48 h)	The examination score of the exercise group was 15% higher than that of the non-exercise group and 20% higher than that of the non-rest group The knowledge retention rate of the exercise group was higher than the other two groups.

*RCT* randomized controlled trial, *CES* controlled experimental study, *QES* quasi-experimental study, *F/P* feasibility/pilot study, *SA* single-arm study

### Quality assessment

Methodological quality was generally low. Only one study was rated as good, three studies as moderate, and the remainder as poor on the Downs and Black checklist (Supplementary Table 3). All the studies clearly described the research objective, intervention, and outcomes. However, most studies failed to address attrition or blinding, with only two studies providing attrition information [14, 35] and only one study implementing subject blinding [28]. None of the studies calculated power analyses. Taken together, these findings indicate that the current evidence base is methodologically limited and that the review findings should therefore be interpreted with appropriate caution.

### GRADE analysis

The certainty of evidence was rated as low for classroom attention, classroom engagement, and classroom emotions, and very low for academic performance (Supplementary Table 4). Downgrading was primarily due to the generally low methodological quality of the included studies, substantial heterogeneity in interventions and outcome measures, and mixed findings across several outcomes.

### Effects of classroom physical activity breaks on learning behavior and academic performance

Across the included studies, outcomes were categorized and synthesized based on subtype and direction of effect (Table 3). A total of 11 studies reported on classroom attention, 13 studies on classroom engagement, ten studies on classroom emotion, and six studies on academic performance. Overall, classroom physical activity breaks showed generally favorable effects across learning-related behavioral and emotional outcomes, while findings for academic performance were more variable.

**Table 3:** Strength of Evidence on the Effects of classroom physical activity breaks on learning behaviors and academic performance

Relevant Indicators		Result	Reference	Supporting/total studies (%)
Classroom Attention	Selective attention	**+**	Peiris CL et al., [14]; Niedermeier M et al., [26]	2/4 (50%) ?
		n.s.	Bantoft C et al., [29]	1/4 (25%) †
		**-**	Dupont F et al., [32]	1/4 (25%) †
	Sustained attention	**+**	Paulus M et al., [22] ; Hayes SM et al., [25]; Jerome M et al., [34]; Pastor-Vicedo JC et al., [27]; Fenesi B et al., [23]	5/7 (71.4%) ‡
		n.s.	Bantoft C et al., [29]; Grosprêtre S et al., [33]	2/7 (28.6%) †
Classroom engagement	Behavioral engagement	**+**	Hayes SM et al., [25]; Jerome M et al., [34]	2/3 (66.7%) ?
		n.s.	Grosprêtre S et al., [33]	1/2 (33.3%) ?
	Emotional engagement	**+**	Paulus M et al., [22]; Hayes SM et al., [25); Jerome M et al., [34]; Clement KA et al., [31]; Keating R et al., [13]; Blasche G et al., [17]	6/7 (85.7%) ‡
		n.s.	Pilcher JJ et al., [39]	1/7 (14.3%) †
	Cognitive engagement	**+**	Yu Q et al., [28]; Keating R et al., [13]	2/2 (NA)
	Agentic engagement	**+**	Hayes SM et al., [25]	1/1 (NA)
Classroom emotion	Positive emotion	**+**	Paulus M et al., [22]; Peiris CL et al., [14]; Hayes SM et al., [25]; Maeda H et al., [36]; Niedermeier M et al., [26] ; Ferrer ME et al., [24]	6/7 (85.7%) ‡
		n.s.	Pilcher JJ et al., [39]	1/7 (14.3%) †
	Negative emotion	**+**	Grosprêtre S et al., [33]	1/2 (NA)
		**-**	Dupont F et al., [32]	1/2 (NA)
Academic performance		**+**	Fenesi B et al., [23]; Bastien Tardif C et al., [30]; Yu Q et al., [28]; Dupont F et al., [32]	4/6 (66.7%) ?
		n.s.	Joubert L et al., [35]; Pilcher JJ et al., [39]	2/6 (33.3%) ?

*NA* Not available

**+**, positive significant effects; **-**, negative significant effect; n.s., non-significant differences; †, weak evidence; ? inconclusive evidence; ‡, strong evidence

### Effects on classroom attention

Classroom attention outcomes were further divided into selective attention and sustained attention. Four studies investigated selective attention, with two reporting significant improvements [14, 26], one showing no effect [29], and one reporting a negative outcome [32], yielding unclear evidence strength (50%). Specifically, students engaging in moderate-intensity cycling exhibited significantly lower visual attention than those in the low-intensity group and even lower than the sedentary group, potentially attributable to the greater attentional demands of motor control during moderate-intensity cycling, which reduces the cognitive resources available for academic tasks [32]. Evidence for sustained attention was relatively stronger. Seven studies addressed sustained attention, five of which found significant improvements [22, 23, 25, 27, 34] and two reported no effect [29, 33], resulting in relatively strong evidence strength (71.4%). Improvements were observed across several classroom-based activity formats, including standing breaks, active workstations, teacher-guided movement, student-initiated activities, and short classroom activity breaks. When students were allowed to choose from active workstations (such as stability balls, cycling desks, or standing desks), there were no significant changes in attention or teacher-student interaction for most students. However, instructors consistently reported that active workstations significantly reduce students’ off-task behaviors, such as chatting or using mobile phones during class [33].

### Effects on classroom engagement

Classroom engagement is a multidimensional construct that reflects students’ engagement with classroom experiences, typically encompassing behavioral, emotional, cognitive, and agentic engagement. Three studies investigated behavioral engagement, with two showing improvements [25, 34] and one reporting no effect [33], yielding unclear evidence (66.7%) in this review. Cognitive and agentic engagement were each examined in fewer than three studies. Although the available findings suggested positive effects, the strength could not be determined [13, 25, 28]. Notably, seven studies investigated emotional engagement, six of which reported significant improvements [13, 17, 22, 25, 31, 34], whereas one reported no effect [38], resulting in relatively strong evidence strength (85.7%). The positive effects of classroom physical activity breaks on emotional engagement were mainly reflected in increased enjoyment, learning motivation, peer interaction, classroom interactivity, and perceived emotional experience. These benefits were more frequently reported after student-led exercise, group-based interaction, or game-like active breaks. In contrast, the use of cycling desks showed no significant difference in motivation or morale compared to traditional desks.

### Effects on classroom emotion

Academic emotions are recognized as a critical determinant of learning outcomes in educational psychology [39]. Positive emotions enhance task engagement and foster adaptive coping strategies, whereas negative emotions may impair learning performance by depleting working memory, attentional capacity, and energy levels [40, 41]. Regarding the impact of classroom physical activity breaks, seven studies explored positive emotions, with six reporting significant improvements [14, 22, 24–26, 36] and one showing no effect [38], resulting in relatively strong evidence strength (85.7%). Evidence for negative emotions was limited and inconsistent. Two studies investigated negative emotions, with one indicating reductions [33] and one indicating an increase [32]. Specifically, physical activity breaks in classroom lectures enhance students’ enjoyment and well-being [14, 22], reduce the frequency of mobile phone use, and alleviate negative emotional states such as stress and boredom [33, 34], compared to traditional sedentary classroom formats. However, the use of tabletop stationary bikes was perceived by several students as “strenuous” and “distracting”, potentially due to insufficient adaptation to the novel active breaks-based learning model [38].

### Effects on academic performance

Six studies assessed academic performance, with four studies indicating improvements [23, 28, 30, 32] and two showing no effects [35, 38], also yielding unclear evidence (66.7%). Compared with passive rest or no rest, physical activity breaks significantly improve students’ memory performance, speaker clarity ratings, and material comprehension. However, studies evaluating active workstations found no significant differences in academic exam scores between users of these workstations and those using traditional desks, suggesting that active workstations do not impair cognitive performance [30, 35]. Therefore, the integration of active workstations into educational settings is considered both acceptable and feasible, offering a strategy for prolonged sedentary behavior and receiving broad student support [13, 36].

## Discussion

Classroom physical activity breaks, or dynamic learning environments, significantly improved students’ attention, positive emotions, and classroom engagement. Fewer studies reported positive effects on academic performance, although these findings were less consistent. This pattern may be partly explained by the immediate restorative effects of brief movement during prolonged sedentary learning. Classroom attention is essential for academic success, but vulnerable to internal and external disruptions [42]. During extended lectures, students may experience increased mental workload, heightened distractibility, reduced attentional control, and lower knowledge retention [43]. In this context, classroom physical activity breaks may act as an active recovery strategy by shifting students from prolonged sedentary cognition to light physical movement, thereby reducing fatigue, restoring alertness, and supporting sustained attention.

The benefits observed for engagement and emotional states may further reflect the social and affective value of activity breaks. Student engagement is crucial for effective learning [44]. Actively engaged students are more likely to focus attention, persist through challenges, build relationships with instructors and peers, and foster a strong connection to the school environment. Furthermore, emotional engagement may represent a distinct and complex psychological process that influences academic outcomes and student well-being [45, 46]. Therefore, when designing curricula, educators should consider strategies to foster comprehensive student engagement by cultivating a supportive and dynamic learning environment.

Among college students, positive academic emotions appear to exert a more substantial influence on learning outcomes than negative academic emotions [39]. Multiple studies highlight the importance of incorporating academic emotion regulation into classroom instructional design, such as creating supportive learning environments and adopting innovative pedagogical strategies [47, 48]. However, the mood-enhancing effects of classroom physical activity breaks may be time-dependent. Previous evidence indicates that acute exercise (especially low-intensity activities lasting no more than 15 min) has a significant impact on subjective vitality and positive affect, with effects peaking immediately post-exercise and gradually diminishing thereafter [49]. Similarly, incorporating structured exercise breaks into university lectures significantly boosts students’ vitality and alleviates fatigue, with effects sustained for up to 20 min [17]. However, another study reported that emotional improvements diminish within 30 min [26], indicating a transient effect and highlighting the importance of timing in optimizing emotional outcomes during instruction. These findings suggest that the timing of activity breaks should be considered carefully, especially when they are used to counter fatigue or declining affect during prolonged instruction.

Evidence for academic performance was less consistent than that for attention, engagement, or positive emotional states. Academic performance is a distal outcome influenced by multiple intermediate processes, including attention, motivation, emotional state, fatigue, and knowledge retention [50]. The effort-recovery model emphasizes the dynamic and reversible nature of fatigue, proposing that physical and mental fatigue caused by task demands can be alleviated through restorative activities, such as appropriate rest or physical exercise [51]. Nonetheless, if recovery is inadequate or task demands persistently exceed individual adaptability, accumulated fatigue may impair short-term performance (e.g., learning efficiency) and long-term health outcomes (e.g., chronic stress) [52]. When compensatory mechanisms fail, classroom physical activity breaks can serve as an active recovery strategy by shifting the task mode from sedentary cognition to light physical activity, thereby mitigating fatigue accumulation, enhancing academic performance, and promoting knowledge retention [17, 53]. Additionally, academic performance was assessed using heterogeneous tools, including short-term memory tasks, delayed quizzes, course-specific progress monitoring tools, and examination scores. Course-specific measures may be more sensitive to subtle classroom-level changes than broader standardized assessments, which may partly explain the inconsistent findings for academic performance.

Several physiological mechanisms have been proposed to explain the beneficial effects of classroom physical activity breaks on learning behaviors and academic performance. One possible mechanism involves the upregulation of brain-derived neurotrophic factor (BDNF) expression, which is associated with improvements in cardiorespiratory fitness and has been closely linked to academic performance [54]. BDNF plays a critical role in various neurocognitive processes, including promoting synaptic plasticity in the hippocampus, regulating the formation of neuronal circuits, modulating emotional decision-making, and facilitating long-term memory consolidation [55]. In addition to its neurotrophic effects, physical activity has been shown to produce acute increases in arousal, alertness, and perceived energy levels, potentially due to enhanced brain activity during movement [56]. Physical activity also enhances neurotransmitter dynamics, increasing the synapse availability of dopamine, norepinephrine, and serotonin, thereby supporting attentional arousal and cognitive engagement [57]. Anxiety also negatively correlates with academic performance. Students with high trait anxiety tend to demonstrate poorer academic performance [58]. However, classroom physical activity breaks may be regarded as an effective intervention against stress response by reducing sympathetic nervous system activity and enhancing parasympathetic modulation [59]. Furthermore, physical activity breaks have been found to enhance executive function, improve retinal vascular diameter, increase cortical activation, and strengthen brain network connectivity, highlighting the neurocognitive benefits of classroom physical activity breaks in sedentary learning environments [28]. In summary, classroom physical activity breaks exert positive effects on learning behaviors and academic performance through various interconnected physiological adaptations, contributing to improved learning conditions and enhanced academic efficiency among college students.

### Limitations and recommendations

This study conducted a comprehensive literature search and systematic review of the relationship between classroom physical activity breaks and college students’ learning behaviors and academic performance. However, several limitations should be acknowledged. First, the exclusion of non-English and grey literature may have introduced publication bias. Second, substantial heterogeneity in study design, intervention protocols, and outcome measures limited causal inference. Third, the overall methodological quality of the included studies was generally low. Finally, most studies primarily reflected students’ and teachers’ perspectives, neglecting other important stakeholders, such as educational administrators. Despite these limitations, this review underscores the disparity between traditional sedentary classrooms and interventions that incorporate physical activity breaks, emphasizing the urgent need for well-designed programs to enhance academic-related outcomes. Numerous studies have noted that due to the demanding nature of curricula, instructors often face difficulties incorporating physical activity breaks without compromising teaching time [18, 60]. Therefore, to improve feasibility, activity breaks should be thematically linked to course content, allowing for greater flexibility based on instructional dynamics. Although the implementation of active workstations (e.g., standing desks) may incur high initial costs, preliminary workplace studies suggest potential long-term economic benefits through reductions in sedentary-related health risks [61]. However, cost-effectiveness evidence in higher education remains limited, necessitating further research to inform policy decisions. Most studies did not report adherence or fidelity metrics, making it difficult to assess implementation quality. Future research should prioritize standardized intervention protocols, teacher training, and adherence monitoring to ensure replicability and scalability.

## Conclusions

Classroom physical activity breaks present a promising strategy for improving academic-related outcomes, particularly emotional engagement, positive academic emotions, and sustained attention. In addition, classroom physical activity breaks may also improve selective attention and behavioral engagement, mitigate negative emotions, and contribute to better academic performance, while further validation is warranted. Furthermore, both students and teachers have expressed willingness to adopt classroom physical activity breaks, believing that these breaks have the potential to support both academic success and overall well-being. Given the prevalence and detrimental impact of sedentary behavior in higher education, greater emphasis should be placed on the implementation and investigation of classroom physical activity breaks.

## Supplementary Information

Supplementary Material 1.

## Data Availability

All data generated or analysed during this study are included in this published article and its supplementary information files.

## Abbreviations

BDNF: Brain-derived neurotrophic factor
CES: Controlled experimental study
F/P: Feasibility/pilot study
GRADE: Grading of Recommendations Assessment, Development and Evaluation
METs: Metabolic equivalents
POMS-SF: Profile of Mood States-Short Form
PRISMA: Preferred Reporting Items for Systematic Reviews and Meta-Analyses
PROSPERO: International Prospective Register of Systematic Reviews
QES: Quasi-experimental study
RCT: Randomized controlled trial
RPE: Rating of perceived exertion
SA: Single-arm study
SOPLAY: System for Observing Play and Leisure Activity in Youth
VO_2_peak: Peak oxygen uptake
