# Supplementary material for: Effect of classroom physical activity breaks on learning behaviors and academic achievements in university students: a systematic review
Source: Int J Behav Nutr Phys Act. 2026 Jun 20;23:81. doi: 10.1186/s12966-026-01936-7 (PMC13543494; doi:10.1186/s12966-026-01936-7)
Supplement: Supplementary file 1 [file 12966_2026_1936_MOESM1_ESM.docx]

**Table 1 Search strategy**

| **PubMed** | | **Total** |
| --- | --- | --- |
| #1 | "Classroom intervention"[Title/Abstract] OR "class* break*"[Title/Abstract] OR "movement break*"[Title/Abstract] OR "exercise* break*"[Title/Abstract] OR "active learning"[Title/Abstract] OR "active lesson*"[Title/Abstract] OR "classroom physical activity breaks"[Title/Abstract] OR "brain break*"[Title/Abstract] OR "activ* break*"[Title/Abstract] OR "Physical* active learning"[Title/Abstract] OR "activ* workstation*"[Title/Abstract] OR "sit-to-stand desk*"[Title/Abstract] OR "sit-stand desk*"[Title/Abstract] OR "breaks in sitting time"[Title/Abstract] OR "walk* break"[Title/Abstract] OR "height-adjustable desk*"[Title/Abstract] OR "sedentary break*"[Title/Abstract] | 6,694 |
| #2 | "Postsecondary Education"[Title/Abstract] OR "Graduate education"[Title/Abstract] OR Tertiary[Title/Abstract] OR college[Title/Abstract] OR universit*[Title/Abstract] OR "higher education"[Title/Abstract] OR "higher learning"[Title/Abstract] OR "university lecture*"[Title/Abstract] OR "university workshop*"[Title/Abstract] OR "lecture-based"[Title/Abstract] OR "tutorial-based"[Title/Abstract] OR "workshop-based"[Title/Abstract] OR Undergraduate[Title/Abstract] OR "Undergraduate Student"[Title/Abstract] OR "Graduate student"[Title/Abstract] OR "Master student"[Title/Abstract] OR "Doctoral student"[Title/Abstract] OR postgraduate[Title/Abstract] OR "college student"[Title/Abstract] OR "Higher education student"[Title/Abstract] OR "Tertiary student"[Title/Abstract] OR "Academic student"[Title/Abstract] OR "Bachelor student"[Title/Abstract] | 953,874 |
| #3 | Attention[MeSH Terms] OR Cognition[MeSH Terms] OR Emotion[MeSH Terms] OR "Educational status"[Title/Abstract] OR "educational measurement"[Title/Abstract] OR "classroom behavior*"[Title/Abstract] OR Grade[Title/Abstract] OR score[Title/Abstract] OR mark[Title/Abstract] OR "Academic achievement"[Title/Abstract] OR "Scholastic attainment"[Title/Abstract] OR "Academic performance"[Title/Abstract] OR "Educational outcomes"[Title/Abstract] OR "Academic success"[Title/Abstract] OR "Grade point average"[Title/Abstract] OR "Standardi* test scores"[Title/Abstract] OR "test scores"[Title/Abstract] OR "Academic record"[Title/Abstract] OR literacy[Title/Abstract] OR numeracy[Title/Abstract] OR "Academic attent*"[Title/Abstract] OR achievement[Title/Abstract] OR Concentration[Title/Abstract] OR Focus[Title/Abstract] OR "Focused attention"[Title/Abstract] OR "Attentional control"[Title/Abstract] OR "Sustained attention"[Title/Abstract] OR "Selective attention"[Title/Abstract] OR cogniti*[Title/Abstract] OR "executive function"[Title/Abstract] OR "fluid intelligence"[Title/Abstract] OR "Working memory"[Title/Abstract] OR "Problem-solving skills"[Title/Abstract] OR "Reasoning ability"[Title/Abstract] OR Intelligence[Title/Abstract] OR "Classroom participation"[Title/Abstract] OR "Student engagement"[Title/Abstract] OR "Active learning"[Title/Abstract] OR "Student involvement"[Title/Abstract] OR emotion*[Title/Abstract] OR "emotion* regulation"[Title/Abstract] OR "emotion* management"[Title/Abstract] OR "emotion* control"[Title/Abstract] OR "self-control"[Title/Abstract] OR "self-regulation"[Title/Abstract] OR "facial expression"[Title/Abstract] | 4,813,617 |
| #4 | #1 AND #2 AND #3 | 1,756 |

| **Web of Science** | | **Total** |
| --- | --- | --- |
| #1 | AB="Classroom intervention" OR AB="class* break*" OR AB="movement break*" OR AB="exercise* break*" OR AB="active learning" OR AB="active lesson*" OR AB="education outside of the classroom" OR AB="classroom physical activity breaks" OR AB="Mov* and academic learning" OR AB="brain break*" OR AB="activ* break*" OR AB="Physical* active learning" OR AB="activ* workstation*" OR AB="sit-to-stand desk*" OR AB="sit-stand desk*" OR AB="breaks in sitting time" OR AB="walk* break" OR AB="breaking up prolonged sitting" OR AB="height-adjustable desk*" OR AB="sedentary break*" | 16,688 |
| #2 | AB="Postsecondary Education" OR AB="Graduate education" OR AB=Tertiary OR AB=college OR AB=universit* OR AB="higher education" OR AB="higher learning" OR AB="university lecture*" OR AB="university workshop*" OR AB="lecture-based" OR AB="tutorial-based" OR AB="workshop-based" OR AB=Undergraduate OR AB="Undergraduate Student" OR AB="Graduate student" OR AB="Master student" OR AB="Doctoral student" OR AB=postgraduate OR AB="college student" OR AB="Higher education student" OR AB="Tertiary student" OR AB="Academic student" OR AB="Bachelor student" | 1,823,536 |
| #3 | (TS=Attention OR TS=Cognition OR TS=Emotion OR AB="Educational status" OR AB="educational measurement" OR AB="classroom behavior*" OR AB=Grade OR AB=score OR AB=mark OR AB="Academic achievement" OR AB="Scholastic attainment" OR AB="Academic performance" OR AB="Educational outcomes" OR AB="Academic success" OR AB="Grade point average" OR AB="Standardi* test scores" OR AB="test scores" OR AB="Academic record" OR AB="Appraisal of academic achievement" OR AB=literacy OR AB=numeracy OR AB="Academic attent*" OR AB=achievement OR AB=Concentration OR AB=Focus OR AB="Focused attention" OR AB="Attentional control" OR AB="Sustained attention" OR AB="Selective attention" OR AB=cogniti* OR AB="executive function" OR AB="fluid intelligence" OR AB="Working memory" OR AB="Problem-solving skills" OR AB="Reasoning ability" OR AB=Intelligence OR AB="Classroom participation" OR AB="Student engagement" OR AB="Active learning" OR AB="Classroom interaction" OR AB="Student involvement" OR AB=emotion* OR AB="emotion* regulation" OR AB="emotion* management" OR AB="emotion* control" OR AB="self-control" OR AB="self-regulation" OR AB="facial expression") | 13,951,996 |
| #4 | #1 AND #2 AND #3 | 3,729 |

| **Cochrane Library** | | **Total** |
| --- | --- | --- |
| #1 | ("Classroom intervention"):ti,ab,kw OR (class* NEXT break*):ti,ab,kw OR (movement NEXT break*):ti,ab,kw OR (exercise* NEXT break*):ti,ab,kw OR ("active learning"):ti,ab,kw OR (active NEXT lesson*):ti,ab,kw OR ("education outside of the classroom"):ti,ab,kw OR ("classroom physical activity breaks"):ti,ab,kw OR (Mov* and academic learning):ti,ab,kw OR (brain NEXT break*):ti,ab,kw OR (activ* NEXT break*):ti,ab,kw OR (Physical* active learning):ti,ab,kw OR (activ* NEXT workstation*):ti,ab,kw OR (sit-to-stand NEXT desk*):ti,ab,kw OR (sit-stand NEXT desk*):ti,ab,kw OR ("breaks in sitting time"):ti,ab,kw OR (walk* break):ti,ab,kw OR ("breaking up prolonged sitting"):ti,ab,kw OR (height-adjustable NEXT desk*):ti,ab,kw OR (sedentary NEXT break*):ti,ab,kw | 2,030 |
| #2 | ("Postsecondary Education"):ti,ab,kw OR ("Graduate education"):ti,ab,kw OR (Tertiary):ti,ab,kw OR (college):ti,ab,kw OR (universit*):ti,ab,kw OR ("higher education"):ti,ab,kw OR ("higher learning"):ti,ab,kw OR (university NEXT lecture*):ti,ab,kw OR (university NEXT workshop*):ti,ab,kw OR ("lecture-based"):ti,ab,kw OR ("tutorial-based"):ti,ab,kw OR ("workshop-based"):ti,ab,kw OR (Undergraduate):ti,ab,kw OR ("Undergraduate Student"):ti,ab,kw OR ("Graduate student"):ti,ab,kw OR ("Master student"):ti,ab,kw OR ("Doctoral student"):ti,ab,kw OR (postgraduate):ti,ab,kw OR ("college student"):ti,ab,kw OR ("Higher education student"):ti,ab,kw OR ("Tertiary student"):ti,ab,kw OR ("Academic student"):ti,ab,kw OR ("Bachelor student"):ti,ab,kw | 120,241 |
| #3 | (Attention):ti,ab,kw OR (Cognition):ti,ab,kw OR (Emotion):ti,ab,kw OR ("Educational status"):ti,ab,kw OR ("educational measurement"):ti,ab,kw OR (classroom NEXT behavior*):ti,ab,kw OR (Grade):ti,ab,kw OR (score):ti,ab,kw OR (mark):ti,ab,kw OR ("Academic achievement"):ti,ab,kw OR ("Scholastic attainment"):ti,ab,kw OR ("Academic performance"):ti,ab,kw OR ("Educational outcomes"):ti,ab,kw OR ("Academic success"):ti,ab,kw OR ("Grade point average"):ti,ab,kw OR (Standardi* test scores):ti,ab,kw OR ("test scores"):ti,ab,kw OR ("Academic record"):ti,ab,kw OR ("Appraisal of academic achievement"):ti,ab,kw OR (literacy):ti,ab,kw OR (numeracy):ti,ab,kw OR (Academic NEXT attent*):ti,ab,kw OR (achievement):ti,ab,kw OR (Concentration):ti,ab,kw OR (Focus):ti,ab,kw OR ("Focused attention"):ti,ab,kw OR ("Attentional control"):ti,ab,kw OR ("Sustained attention"):ti,ab,kw OR ("Selective attention"):ti,ab,kw OR (cogniti*):ti,ab,kw OR ("executive function"):ti,ab,kw OR ("fluid intelligence"):ti,ab,kw OR ("Working memory"):ti,ab,kw OR ("Problem-solving skills"):ti,ab,kw OR ("Reasoning ability"):ti,ab,kw OR (Intelligence):ti,ab,kw OR ("Classroom participation"):ti,ab,kw OR ("Student engagement"):ti,ab,kw OR ("Active learning"):ti,ab,kw OR ("Classroom interaction"):ti,ab,kw OR ("Student involvement"):ti,ab,kw OR (emotion* regulation):ti,ab,kw OR (emotion* management):ti,ab,kw OR (emotion* control):ti,ab,kw OR (self-control):ti,ab,kw OR (self-regulation):ti,ab,kw OR ("facial expression"):ti,ab,kw | 617,747 |
| #4 | #1 AND #2 AND #3 | 315 |

**Table 2 PRISMA checklist**

| **Section and Topic** | **Item** | **Checklist item** | **Location where item is reported** |
| --- | --- | --- | --- |
| **TITLE** | | |  |
| Title | 1 | Identify the report as a systematic review. | Title page |
| **ABSTRACT** | | |  |
| Abstract | 2 | See the PRISMA 2020 for Abstracts checklist. | Abstract |
| **INTRODUCTION** | | |  |
| Rationale | 3 | Describe the rationale for the review in the context of existing knowledge. | Background, paragraphs 1-2 |
| Objectives | 4 | Provide an explicit statement of the objective(s) or question(s) the review addresses. | Background, final paragraph |
| **METHODS** | | |  |
| Eligibility criteria | 5 | Specify the inclusion and exclusion criteria for the review and how studies were grouped for the syntheses. | Methods, Eligibility criteria; Table 1; Table 3 |
| Information sources | 6 | Specify all databases, registers, websites, organisations, reference lists, and other sources searched or consulted to identify studies. Specify the date when each source was last searched or consulted. | Methods, Search strategy; Methods, Study selection and data extraction |
| Search strategy | 7 | Present the full search strategies for all databases, registers, and websites, including any filters and limits used. | Methods, Search strategy; Supplementary Table 1 |
| Selection process | 8 | Specify the methods used to decide whether a study met the inclusion criteria of the review, including how many reviewers screened each record and each report retrieved, whether they worked independently, and, if applicable, details of automation tools used in the process. | Methods, Eligibility criteria; Methods, Study selection and data extraction; Figure 1 |
| Data collection process | 9 | Specify the methods used to collect data from reports, including how many reviewers collected data from each report, whether they worked independently, any processes for obtaining or confirming data from study investigators, and if applicable, details of automation tools used in the process. | Methods, Study selection, and data extraction |
| Data items | 10a | List and define all outcomes for which data were sought. Specify whether all results that were compatible with each outcome domain in each study were sought (e.g. for all measures, time points, analyses), and if not, the methods used to decide which results to collect. | Methods, Coding and classification of outcomes; Table 1; Table 3 |
|  | 10b | List and define all other variables for which data were sought (e.g. participant and intervention characteristics, funding sources). Describe any assumptions made about any missing or unclear information. | Methods, Study selection, and data extraction; Table 2 |
| Study risk of bias assessment | 11 | Specify the methods used to assess risk of bias in the included studies, including details of the tool(s) used, how many reviewers assessed each study and whether they worked independently, and if applicable, details of automation tools used in the process. | Methods, Quality assessment, and certainty of evidence; Supplementary Table 2 |
| Effect measures | 12 | Specify for each outcome the effect measure(s) (e.g. risk ratio, mean difference) used in the synthesis or presentation of results. | Methods, Coding and classification of outcomes; Results, Effects of classroom physical activity breaks on learning behavior and academic performance; Table 3 |
| Synthesis methods | 13a | Describe the processes used to decide which studies were eligible for each synthesis (e.g. tabulating the study intervention characteristics and comparing against the planned groups for each synthesis (item #5)). | Methods, Eligibility criteria; Methods, Coding and classification of outcomes; Table 2; Table 3 |
|  | 13b | Describe any methods required to prepare the data for presentation or synthesis, such as handling of missing summary statistics, or data conversions. | Not applicable; no data conversions were performed because the review used narrative synthesis and direction-of-effect coding. |
|  | 13c | Describe any methods used to tabulate or visually display results of individual studies and syntheses. | Methods, Coding and classification of outcomes; Results, Table 2; Table 3 |
|  | 13d | Describe any methods used to synthesize results and provide a rationale for the choice(s). If meta-analysis was performed, describe the model(s), method(s) to identify the presence and extent of statistical heterogeneity, and software package(s) used. | Methods, Coding and classification of outcomes; Results, Effects by outcome domain; Table 3. No meta-analysis was conducted because of heterogeneity in interventions and outcome measures. |
|  | 13e | Describe any methods used to explore possible causes of heterogeneity among study results (e.g. subgroup analysis, meta-regression). | Results, Study characteristics; Discussion; Limitations, and recommendations. Heterogeneity was explored narratively by intervention format, study design, and outcome domain. |
|  | 13f | Describe any sensitivity analyses conducted to assess robustness of the synthesized results. | Not applicable; no formal sensitivity analysis was conducted because meta-analysis was not performed. |
| Reporting bias assessment | 14 | Describe any methods used to assess risk of bias due to missing results in a synthesis (arising from reporting biases). | Not formally assessed; the small number of studies per outcome and narrative synthesis limited assessment of reporting bias. Addressed as a limitation in Limitations and recommendations. |
| Certainty assessment | 15 | Describe any methods used to assess certainty (or confidence) in the body of evidence for an outcome. | Methods, Quality assessment and certainty of evidence; Results, GRADE analysis; Supplementary Table 3 |
| **RESULTS** | | |  |
| Study selection | 16a | Describe the results of the search and selection process, from the number of records identified in the search to the number of studies included in the review, ideally using a flow diagram. | Methods, Study selection and data extraction; Results, Study characteristics; Figure 1 |
|  | 16b | Cite studies that might appear to meet the inclusion criteria, but which were excluded, and explain why they were excluded. | Not applicable; excluded full-text studies are summarized in the study selection process/PRISMA flow diagram. No separate excluded-study table was prepared. |
| Study characteristics | 17 | Cite each included study and present its characteristics. | Results, Study characteristics; Table 2 |
| Risk of bias in studies | 18 | Present assessments of risk of bias for each included study. | Results, Quality assessment; Supplementary Table 2 |
| Results of individual studies | 19 | For all outcomes, present, for each study: (a) summary statistics for each group (where appropriate) and (b) an effect estimate and its precision (e.g. confidence/credible interval), ideally using structured tables or plots. | Results, outcome-specific subsections; Table 2; Table 3 |
| Results of syntheses | 20a | For each synthesis, briefly summarise the characteristics and risk of bias among contributing studies. | Results, Quality assessment; Results, GRADE analysis; Table 3; Supplementary Tables 2-3 |
|  | 20b | Present results of all statistical syntheses conducted. If meta-analysis was done, present for each the summary estimate and its precision (e.g. confidence/credible interval) and measures of statistical heterogeneity. If comparing groups, describe the direction of the effect. | Results, Effects by outcome domain; Table 3. No statistical synthesis/meta-analysis was conducted. |
|  | 20c | Present results of all investigations of possible causes of heterogeneity among study results. | Results, Study characteristics; Results, Effects by outcome domain; Discussion |
|  | 20d | Present results of all sensitivity analyses conducted to assess the robustness of the synthesized results. | Not applicable; no sensitivity analysis was conducted. |
| Reporting biases | 21 | Present assessments of risk of bias due to missing results (arising from reporting biases) for each synthesis assessed. | Not formally assessed; narrative synthesis and small numbers of studies per outcome limited assessment. |
| Certainty of evidence | 22 | Present assessments of certainty (or confidence) in the body of evidence for each outcome assessed. | Results, GRADE analysis; Supplementary Table 3 |
| **DISCUSSION** | | |  |
| Discussion | 23a | Provide a general interpretation of the results in the context of other evidence. | Discussion |
|  | 23b | Discuss any limitations of the evidence included in the review. | Discussion; Limitations and recommendations |
|  | 23c | Discuss any limitations of the review processes used. | Limitations and recommendations |
|  | 23d | Discuss implications of the results for practice, policy, and future research. | Discussion; Limitations and recommendations; Conclusions |
| **OTHER INFORMATION** | | |  |
| Registration and protocol | 24a | Provide registration information for the review, including register name and registration number, or state that the review was not registered. | Methods, Study selection, and data extraction (PROSPERO registration number CRD420251047553) |
|  | 24b | Indicate where the review protocol can be accessed, or state that a protocol was not prepared. | Not applicable; the protocol was not separately prepared beyond PROSPERO registration. |
|  | 24c | Describe and explain any amendments to information provided at registration or in the protocol. | Not applicable; no amendments to the registered review information were reported. |
| Support | 25 | Describe sources of financial or non-financial support for the review, and the role of the funders or sponsors in the review. | Declarations, Funding |
| Competing interests | 26 | Declare any competing interests of review authors. | Declarations, Competing interests |
| Availability of data, code and other materials | 27 | Report which of the following are publicly available and where they can be found: template data collection forms; data extracted from included studies; data used for all analyses; analytic code; any other materials used in the review. | Declarations, Availability of data and materials; Supplementary Tables 1-3 |

**Table 3 Results of the quality assessment**

|  | Reporting | External Validity | Internal Validity  (Bias) | Internal Validity  (Confounding) | Power | Total |
| --- | --- | --- | --- | --- | --- | --- |
| Question Numbers | 1-10 | 11-13 | 14-20 | 21-26 | 27 |  |
| Maximum Score | 11 | 3 | 7 | 6 | 1 | 28 |
| Bantoft C *et al.*, 2016 | 4 | 2 | 3 | 1 | 0 | 10 |
| Joubert L *et al.*, 2017 | 7 | 2 | 5 | 4 | 0 | 18 |
| Paulus M *et al.*, 2021 | 6 | 2 | 4 | 2 | 0 | 14 |
| Peiris CL *et al.*, 2021 | 9 | 3 | 5 | 3 | 0 | 20 |
| Pilcher JJ *et al.*, 2017 | 8 | 0 | 3 | 2 | 0 | 13 |
| Hayes SM *et al.*, 2024 | 5 | 0 | 3 | 1 | 0 | 9 |
| Jerome M *et al.*, 2017 | 8 | 2 | 3 | 3 | 0 | 16 |
| Bastien Tardif C *et al.*, 2018 | 5 | 2 | 3 | 1 | 0 | 11 |
| Grosprêtre S *et al.*, 2021 | 5 | 0 | 3 | 1 | 0 | 10 |
| Blasche G *et al.*, 2018 | 5 | 2 | 3 | 1 | 0 | 11 |
| Clement KA *et al.*, 2018 | 4 | 2 | 3 | 1 | 0 | 10 |
| Maeda H *et al.*, 2014 | 5 | 2 | 3 | 1 | 0 | 10 |
| Mnich C *et al*., 2019 | 4 | 3 | 3 | 1 | 0 | 11 |
| Keating R *et al.*, 2020 | 6 | 2 | 2 | 0 | 0 | 10 |
| Niedermeier M *et al.*, 2020 | 8 | 3 | 4 | 3 | 0 | 18 |
| Yu Q *et al.*, 2025 | 5 | 2 | 4 | 2 | 0 | 13 |
| Dupont F *et al.*, 2024 | 5 | 2 | 3 | 1 | 0 | 11 |
| Ferrer ME *et al.*, 2017 | 4 | 0 | 3 | 1 | 0 | 8 |
| Pastor-Vicedo JC *et al.*, 2024 | 5 | 2 | 3 | 2 | 0 | 12 |
| Fenesi B *et al.*, 2018 | 6 | 2 | 3 | 3 | 0 | 14 |

**Table 4 GRADE analysis**

| Outcome | No. | Risk of bias | Inconsistency | Indirectness | Imprecision | Publication bias | Summary of findings | Certainty of evidence |
| --- | --- | --- | --- | --- | --- | --- | --- | --- |
| Classroom attention | 11 | Serious^a,b^ | Serious^c^ | Not serious | Serious^d^ | NA^e^ | For selective attention, 2/4 studies reported a significant improvement, 1/4 reported no significant effect, and 1/4 reported a negative effect. | ⊕⊕⊖⊖ |
| Classroom engagement | 12 | Serious^a,b^ | Serious^c^ | Not serious | Serious^d^ | NA^e^ | Behavioral engagement showed mixed findings (2/3 positive; 1/3 no effect). Emotional engagement showed generally favorable findings (5/6 positive; 1/6 no effect). Cognitive (2) and agentic (1) engagement were each assessed in fewer than three studies, precluding firm conclusions. | ⊕⊕⊖⊖ |
| Classroom emotions | 10 | Serious^a,b^ | Serious^c^ | Not serious | Serious^d^ | NA^e^ | Positive emotions showed generally favorable findings (6/7 positive; 1/7 no effect). Negative emotions showed mixed findings (2/3 reductions; 1/3 increase). | ⊕⊕⊖⊖ |
| Academic performance | 6 | Serious^a,b^ | Serious^c^ | Serious^f^ | Serious^d^ | NA^e^ | Findings were mixed: 4/6 studies reported significant improvements, whereas 2/6 found no significant effect. | ⊕⊖⊖⊖ |

a. Downgraded for risk of bias: most included studies were rated as poor methodological quality according to the Downs and Black checklist.

b. Downgraded for risk of bias: methodological limitations included inadequate blinding, insufficient reporting of attrition, and lack of statistical power analyses.

c. Downgraded for inconsistency: substantial heterogeneity was present across studies in terms of intervention type, duration, intensity, comparator conditions, and outcome measures, and several outcomes showed mixed directions of effect.

d. Downgraded for imprecision: many included studies had small sample sizes, and pooled effect estimates were unavailable because the review was based on narrative synthesis.

e. Publication bias could not be reliably assessed because the number of studies per outcome was limited and the included studies were heterogeneous.
